# Effects of plain packaging, warning labels, and taxes on young people’s predicted sugar-sweetened beverage preferences: an experimental study

**DOI:** 10.1186/s12966-016-0421-7

**Published:** 2016-09-01

**Authors:** Tessa Bollard, Ninya Maubach, Natalie Walker, Cliona Ni Mhurchu

**Affiliations:** 1 Auckland District Health Board, Auckland City Hospital Nutrition, Auckland, New Zealand; 2 Department of Marketing, University of Otago, Dunedin, New Zealand; 3 National Institute for Health Innovation, University of Auckland, Private Bag 92019, Auckland, New Zealand

**Keywords:** Sugar sweetened beverage, Plain packaging, Nutrition labels, Tax, Experiment

## Abstract

**Background:**

Consumption of sugar-sweetened beverages (SSBs) is associated with increased risk of obesity, diabetes, heart disease and dental caries. Our aim was to assess the effects of plain packaging, warning labels, and a 20 % tax on predicted SSB preferences, beliefs and purchase probabilities amongst young people.

**Methods:**

A 2 × 3 × 2 between-group experimental study was conducted over a one-week period in August 2014. Intervention scenarios were delivered, and outcome data collected, via an anonymous online survey. Participants were 604 New Zealand young people aged 13–24 years who consumed soft drinks regularly. Participants were randomly allocated using a computer-generated algorithm to view one of 12 experimental conditions, specifically images of branded versus plain packaged SSBs, with either no warning, a text warning, or a graphic warning, and with or without a 20 % tax. Participant perceptions of the allocated SSB product and of those who might consume the product were measured using seven-point Likert scales. Purchase probabilities were measured using 11-point Juster scales.

**Results:**

Six hundred and four young people completed the survey (51 % female, mean age 18 (SD 3.4) years). All three intervention scenarios had a significant negative effect on preferences for SSBs (plain packaging: *F* (6, 587) = 54.4, *p* <0.001; warning label: *F* (6, 588) = 19.8, *p* <0.001; 20 % tax: *F* (6, 587) = 11.3, *p* <0.001). Plain packaging and warning labels also had a significant negative impact on reported likelihood of purchasing SSB’s (*p* = <0.001). A 20 % tax reduced participants’ purchase probability but the difference was not statistically significant (*p* = 0.2).

**Conclusions:**

Plain packaging and warning labels significantly reduce young people’s predicted preferences for, and reported probability of purchasing, SSBs.

## Background

Consumption of sugar-sweetened beverages (SSBs) is associated with increased risk of obesity, diabetes, heart disease, and dental caries [1–4]. Interventions such as effective nutrition labelling, marketing restrictions, and taxes have been proposed as strategies to improve diets, tackle obesity, and reduce healthcare costs [5]. Research on another ‘dangerous consumption’, tobacco, has shown plain packaging, warning labels and excise taxes are all highly effective ways to reduce consumption [6–10]. A number of countries have levied taxes on SSBs (e.g. Mexico, France, Hungary, and several states in the United States of America) and emerging evidence suggests that they are effective [11]. For example, evaluation of a recently introduced 10 % tax on SSBs in Mexico shows an average 6 % decline in purchases of taxed beverages in 2014 relative to the counterfactual [12].

Less is known however regarding the effects of warning labels and plain packaging on diets. In Finland, warning labels are mandatory on foods high in salt and their introduction led to a decrease in availability of high salt foods [13], thus reportedly contributing to reductions in population salt intakes over time [14]. Sales of diet soft drinks were also significantly reduced after introduction of saccharin warning labels in the USA [15], and from December 2015, New York City chain restaurants have had to place warnings on high salt menu items [16]. Experimental research suggests that warning labels on SSBs reduce parents’ intentions to purchase SSBs for their children [17].

In 2014, a bill was proposed for introduction of warning labels on SSBs in California, although it was subsequently rejected [18]. The city of San Francisco has however approved the warning label, “*WARNING*: *Drinking beverages with added sugar*(*s*) *contributes to obesity*, *diabetes*, *and tooth decay*” on advertisements for SSBs [19].

Although a number of studies have shown that branding influences children’s food preferences [20, 21], the effects of plain packaging and warning labels on SSB preferences and potential consumption are unknown. Therefore we undertook an online experiment to assess the effects of plain packaging, warning labels (text and graphic), and a 20 % tax on predicted product preferences, beliefs and SSB purchase probabilities amongst young people in New Zealand. The tax scenario was included as a reference comparator for the plain packaging and warning label intervention scenarios.

## Methods

### Study design

A 2 (branded packaging, unbranded packaging) × 3 (no warning label, text warning label, graphic warning label) × 2 (no tax, 20 % tax) between-group experimental study design was used, with the intervention scenarios delivered online and outcome data collected entirely via online survey. The study was undertaken in New Zealand over one week in August 2014. Ethical approval was received from the University of Auckland Human Participants Ethics Committee on August 1st 2014 (reference number 012200) and all study participants provided informed consent.

### Recruitment

People were eligible for inclusion in the study if they reported consuming SSB in the previous two months, and were aged between 13–24 years. Recruitment of 16–24 year olds was undertaken by a market research company, Research Now, which invited a random sample of their panel members aged 16 or older to participate in the study. Younger participants (aged 13–16 years) were additionally recruited via parents who were panel members. Recruitment continued until at least 600 eligible respondents consented to take part and were randomised. All participants received a nominal monetary reward (NZ$1, equivalent to US$0.72, €0.64 on 20 May 2016) for 16–24 year olds, and NZ$3, (US$2.17, €1.93) for parents who consented to their 13–16 year old children participating) on completion of the study.

### Interventions

**Fig. 1:**
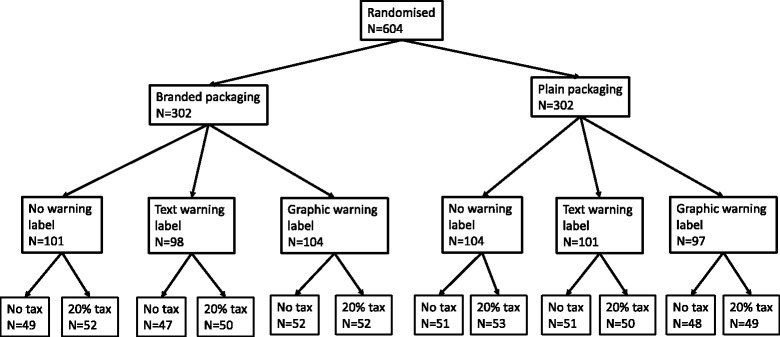
Experimental treatment conditions

Participants were randomly allocated using a computer-generated algorithm to view one of 12 experimental conditions, specifically images of branded versus plain packaged SSB cans, with either no warning, a text warning, or a graphic warning, and price with or without a 20 % tax (Fig. 1). Images were of a well-known carbonated SSB, and the same brand name was used throughout. The plain packaging image used the same colour as that regulated under Australia’s tobacco plain packaging laws, ‘Pantone 448 C’. The brand name was written in size 14, bold Arial font.

The text warning was presented in an orange-coloured octagon and covered approximately one eighth of the front side of the can. It stated “*WARNING*: *high sugar content*” in size 6, bold Arial font. The format was based on that of the Chilean warning labels for foods high in energy, sodium, saturated fat or sugar to be implemented in July 2016 [22]. The graphic warning label was modelled on graphic tobacco health warnings currently used in New Zealand, and displayed an image of dental caries with the accompanying message “*WARNING*: *consuming beverages with added sugar contributes to tooth decay*”. Tooth decay was chosen as the health outcome because there is a strong relationship between sugar consumption and dental caries [23], and it is a more immediate consequence of SSB consumption than obesity. As with the text-only warning label, the pictorial label covered approximately one eighth of the soft drink can’s face. The graphic part of the warning made up half of the label and the text took up the other half (size 6, bold Arial font for the ‘*WARNING*’ and size 4, bold Arial font for the rest of the message).

The tax/no tax scenarios were operationalised by presenting the usual (average) price of a can of the displayed SSB (NZ$2.00, equivalent to US$1.44, €1.29) or the price of a can with 20 % tax applied (NZ$2.40, US$1.73, €1.55) directly below the images.

### Data collection and outcome measures

Prior to the survey, data were collected on participants’ key demographic characteristics, usual consumption of common foods and beverages, and frequency of consumption of a range of beverages including carbonated drinks, energy drinks, milk, and juices. These also served as screening questions to ensure respondents were in the target age range and had consumed SSBs in the previous two months.

Following randomisation, participants viewed the allocated image, and used a seven-point rating scale to answer questions regarding attitudes and predicted product preferences. Specific semantic differential attitude statements assessed whether participants believed the displayed SSB was: expensive/cheap, unattractive/attractive, low quality/high quality, uncool/cool, unhealthy/healthy, and tasted bad or good. As adolescents and young adults often consume SSBs in public settings, and brands are used to communicate aspects of consumers’ identities [24], we also asked questions regarding their perceptions of a peer if they were drinking from the can displayed. Perceptions were measured using four semantic differential questions anchored by: boring/interesting, unpopular/popular, unfashionable/fashionable, and old/young.

The 11-point Juster Scale was used to measure the “in the moment” probability of purchasing the displayed drink if it were one of the options available at a convenience store, where 0 represented “no chance or almost no chance”, 5 represented “fairly good possibility”, and 10 represented “certain or practically certain” [9, 25]. Five-point Likert scales were used to measure participants’ attitudes towards proposed implementation of warning labels and taxes on SSBs. Two versions of the survey were used, one for the young adult group (17–24 years), and another, with simpler language, targeted to the adolescent group (13–16 years).

### Sample size and analysis

Assuming a standard deviation of 1.0, power calculations indicated that a sample size of 600 participants (50 participants per experimental scenario) would provide 80 % power to detect a minimum one-point difference in purchase probability on the Juster scale.

Independent sample *t*-tests were used to assess differences in mean predicted preference scores as a result of packaging or taxes, and a one-way Analysis Of Variance (ANOVA) was used to assess differences in product preferences as a result of warning labels. A three-way ANOVA was conducted to determine whether the three experimental scenarios were associated with significant effects on participants’ likelihood to buy SSBs. Multivariate Analyses Of Variance (MANOVA) were undertaken to assess the relationships between the interventions.

## Results

Six hundred and four panel members responded to the study invitation, met study inclusion criteria, and completed the online survey. Three hundred and three were recruited directly from the panel (age range 16–24 years) and 301 were recruited via parent members (age range 13–16 years). All questions were completed by all participants. Key demographic characteristics are detailed in Table 1. Fifty one percent of the sample was female, and mean age was 18.4 years (SD 3.4). Participants were predominantly of New Zealand European ethnicity (77 %) with fewer identifying as Maori, Pacific or other ethnicities. More than half the survey participants (*n* = 350, 58 %) reported consuming SSBs between one and three times per month, while 49 (8.1 %) consumed SSB at least once a day.

**Table 1:** Demographic characteristics of study sample

Demographic variable	n = 604
Gender, n (%)	
Female	308 (51.0 %)
Male	296 (49.0 %)
Age, mean (SD)	18.4 (3.4)
Ethnicity, n (%)^a^	
NZ European	464 (76.8 %)
Maori	68 (11.3 %)
Pacific	17 (2.8 %)
Chinese	43 (7.1 %)
Indian	21 (3.5 %)
Other	64 (10.6 %)
Don’t Know	3 (0.5 %)
Highest level of education, n (%)	
Intermediate	35 (5.8 %)
High school	307 (49.3 %)
Tertiary	171 (28.3 %)
Other	10 (1.5 %)
Not currently studying/training	99 (16.4 %)
Employment, n (%)	
Casual	125 (20.7 %)
Part time	121 (20.0 %)
Full time	60 (9.9 %)
Not working	308 (51.0 %)
Weekly income, n (%)	
< $100	143 (23.7 %)
$100-$199	60 (9.9 %)
$200-$399	34 (5.6 %)
$400-$599	24 (4.0 %)
$600-$799	26 (4.3 %)
> $800	8 (1.3 %)
Living Situation, n (%)	
Lives with parents or other family	443 (73.5 %)
Lives in shared flat	109 (18.1 %)
Lives in student hostel	9 (1.5 %)
Lives in own home	25 (4.1 %)
Other	17 (2.8 %)
Pocket Money (%)	
No	370 (61.4 %)
Yes	185 (30.7 %)
Receives money for living costs	48 (8.0 %)
Usual frequency of SSB beverage consumption, n (%)	
Less than once a month	87 (14.4 %)
1-3 times per month	350 (58.0 %)
3-4 times per month	82 (13.6 %)
5-6 times per month	36 (6.0 %)
Once a day	29 (4.8 %)
Twice or more a day	20 (3.3 %)

^a^Survey participants could choose more than one ethnicity so total exceeds 100 %

### Impact of interventions on product preferences

Compared with the control of a branded SSB can with no warning label or tax applied, all three interventions had a significant negative effect on predicted preferences for SSBs (plain packaging: *F* (6, 587) = 54.4, *p* <0.001; warning label: *F* (6, 588) = 19.8, *p* <0.001; and 20 % tax: *F* (6, 587) = 11.3, *p* <0.001) (Table 2). Plain packaging led to significantly lower predicted preference scores compared with branded packaging for all measures, except beliefs regarding the healthiness of soft drinks for which there was no difference. As expected, graphic warning labels impacted predicted preferences more negatively than text warning labels. Compared to non-tax conditions, a 20 % tax was associated with perceptions of the product as more expensive (*p* = <0.001), tasting worse (*p* = 0.025), and appearing less ‘cool’ (*p* = 0.034). The tax had no significant effect on other measures however. A significant interaction was found between packaging and warning labels for the attractiveness of the can itself (F (2,592) = 14.9, *p* = <0.001), and between packaging and a 20 % tax for how interesting/boring a typical user was perceived to be (F (1,592) = 4.13, *p* = 0.04).

**Table 2:** Mean predicted preference scores by experimental condition

Preference measure^a^	Plain packaging	Branded packaging	*p*-value	No warning label	Text-only warning label	Pictorial warning label	*p*-value	No tax	20 % tax	*p*-value
	Mean (SD)	Mean (SD)		Mean (SD)	Mean (SD)	Mean (SD)		Mean (SD)	Mean (SD)	
Product attractiveness	2.1 (1.6)	3.9 (1.8)	<0.001	3.7 (2.0)	3.1 (2.0)	2.2 (1.9)	<0.001	3.1 (2.0)	3.0 (1.9)	0.364
Product taste	3.4 (1.8)	5.0 (1.7)	<0.001	4.5 (1.8)	4.2 (2.0)	3.8 (2.0)	<0.001	4.4 (1.9)	4.1 (1.9)	0.025
Product quality	2.6 (1.6)	4.4 (1.5)	<0.001	3.8 (1.8)	3.4 (1.8)	3.2 (1.7)	0.001	3.6 (1.9)	3.5 (1.7)	0.407
Product coolness	2.5 (1.6)	4.2 (1.6)	<0.001	4.0 (1.8)	3.3 (1.7)	2.7 (1.6)	<0.001	3.5 (1.9)	3.2 (1.8)	0.034
Product healthfulness	1.9 (1.2)	1.9 (1.2)	0.546	2.1 (1.3)	1.8 (1.1)	1.8 (1.2)	0.027	1.9 (1.3)	1.9 (1.2)	0.844
Product cost	3.8 (2.1)	3.4 (1.8)	0.042	3.6 (2.0)	3.7 (2.0)	3.5 (1.9)	0.718	4.2 (1.9)	3.0 (1.9)	<0.001
Consumer interest (boring/interesting)	3.2 (1.4)	4.3 (1.1)	<0.001	4.0 (1.3)	3.7 (1.5)	3.5 (1.7)	<0.001	3.8 (1.4)	3.7 (1.3)	0.190
Consumer popularity (unpopular/popular)	3.2 (1.5)	4.4 (1.2)	<0.001	4.0 (1.4)	3.7 (1.5)	3.6 (1.5)	0.002	3.8 (1.5)	3.8 (1.5)	0.699
Consumer fashionableness (unfashionable/fashionable)	2.9 (1.4)	4.2 (1.2)	<0.001	3.9 (1.4)	3.6 (1.5)	3.2 (1.4)	<0.001	3.5 (1.5)	3.6 (1.4)	0.910
Consumer age (old/young)	3.6 (1.8)	3.1 (1.4)	<0.001	3.4 (1.5)	3.4 (1.7)	3.2 (1.7)	0.520	3.4 (1.7)	3.3 (1.6)	0.607

^a^All measures assessed on a scale of 1–7 where 1 was the worst score (least preferable) and 7 was the best (most preferable)

*SD* standard deviation

### Impact of interventions on likelihood to buy SSBs

**Fig. 2:**
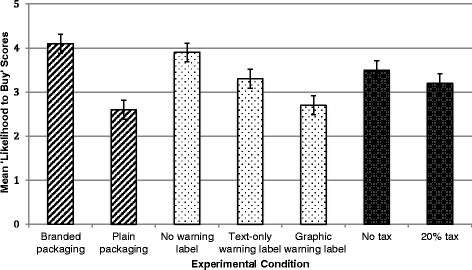
Mean ‘likelihood to buy’ SSB’s by experimental condition. Means are scores on 11-point Juster scale. Error bars represent 95 % confidence intervals around the means

Compared with branded packaging, plain packaging significantly decreased reported likelihood of buying SSBs (2.6 on Juster scale versus 4.1, *p* = <0.001). Text and graphic warning labels also significantly decreased likelihood of buying SSBs compared with no warning label: 3.3 (text warning) and 2.7 (graphic warning) on Juster scale versus 3.9 (no warning), *p* = <0.001. The 20 % tax had no significant effect on probability of purchasing (3.2 versus 3.5, *p* = 0.2) (Fig. 2). The two-way ANOVA revealed that plain packaging (F = 49.0, *p* < 0.001) and warning labels (F = 9.5, *p* < 0.001) had a significant effect on purchase probability, while a 20 % tax did not (F = 1.7, *p* = 0.2).

### Attitudes towards implementation of warning labels and taxes

Two thirds of participants (66 %) agreed/strongly agreed that SSBs should carry a text warning label while half (50 %) agreed/strongly agreed with introduction of graphic warning labels. Fewer participants agreed with application of a tax to SSBs (35 % supported a proposed 10 % price increase, and 26 % supported a proposed 20 % price increase).

## Discussion

To the best of our knowledge this is the first research to examine the effects of plain packaging and warning labels on young people’s predicted preferences for, and likelihood to buy, SSBs. The online experiment suggests that plain packaging and warning labels reduce young people’s preferences for, and reported likelihood to buy, SSBs. Of the experimental conditions examined, plain packaging had the most significant negative impact on predicted product preferences, and was associated with less positive perceptions of those who might consume the product. These results align with findings from studies of plain packaging for tobacco products [6, 9, 26]. Addition of a warning label also had a significant negative impact on predicted SSB preferences. A text-only warning label, recently proposed as a potential public health policy to reduce intake of SSBs [18], reduced perceived product attractiveness, quality and taste, and reduced perceptions of consumer “coolness”. Similar research examining how warning labels on SSBs influenced parents’ choices found they improved parents’ understanding of health harms associated with over-consumption of SSBs and reduced intentions to purchase SSBs for their children [17].

Although graphic warning labels have not yet been proposed for SSBs, this research suggests they would be more effective than text warning labels. Furthermore, warning labels had a greater effect when placed on beverages with plain packaging compared with branded packaging, suggesting that attractive branding and colours on SSBs may reduce effectiveness of warning labels. These results are again consistent with tobacco research [8, 9, 27].

Given the success of tobacco taxes in reducing consumption [7, 10] and emerging evidence of behavioural effects of SSB taxes [12], our finding of only a weak effect of a 20 % tax in this study is unexpected. It is possible however that this was due to how taxes were operationalised in this study where price was displayed in text below the SSB image rather than on a more prominent price label such as those used in supermarkets or convenience stores. The price information may not have been sufficiently visually salient, and the absence of comparator priced products may have limited participants’ ability to judge the relative cost of the products. Our finding may also reflect the relative importance of price and branding to young people. Although smoking research finds that price responsiveness varies inversely with age [28], brand image and social recognition may have an even more powerful influence on adolescents’ preferences and decision making [29].

### Limitations

The online survey method was convenient and quick for participants, and likely had a low level of social desirability bias given no interviewer was present and anonymity was maintained. The study also had a sufficiently large sample size to detect a minimum one-point difference in purchase probability measured on an 11-point Juster scale. Some limitations are however noted. First, the scenarios were hypothetical and respondents did not actually purchase the drinks in question; as such their responses reflect self-predictions rather than actual behaviour [30]. Whilst this limitation applied to all intervention groups equally and thus affects the external validity of our findings rather than internal validity, future experiments could extend this work by presenting participants with real beverages and asking them to purchase their chosen one. Second, findings may not be widely generalizable to the New Zealand population. The survey response rate was low and the proportion of Maori and Pacific participants was lower than in the general New Zealand population of the same age (11 % Maori vs 16 %, and 3 % Pacific vs 8 %), whilst 51 % of survey participants were not employed/working compared to 17 % of 15–24 year olds at a national level. In addition, only one brand and size of SSB was used in this study so further testing would be necessary to determine if findings are generalizable across other brands and products. Finally, the text warning label used in the study was in fact a type of graphic warning because the octagon may have invoked a “Stop Sign” association. This format was chosen because we wanted to base our experiment on labelling interventions being considered or implemented in the real-world where possible. However, it would be useful to repeat the experiment using a simple text warning to determine if effects are similar.

## Conclusions

Plain packaging and warning labels could significantly reduce adolescents’ and young adults’ preferences for and likelihood to purchase SSBs, and may therefore reduce consumption. These labelling measures warrant consideration as part of a comprehensive portfolio of strategies to reduce young people’s SSB intakes and reduce rates of childhood obesity.

## Abbreviations

ANOVA: Analysis of variance
MANOVA: Multivariate analyses of variance
NZ: New Zealand
SD: Standard deviation
SSB: Sugar-sweetened beverage
USA: United States of America
